# *Aspergillus Niger* 129B promotes plant growth by inducing the interaction between SAUR32 and PP2C72

**DOI:** 10.3389/fmicb.2025.1637235

**Published:** 2025-08-26

**Authors:** Juan He, Li Yuan Gao, Yong Ping Luo, Min Chai, Hua Feng Wang, Lu Hong Yang, Kang Zhao, Yushanjiang Maimaiti, Yuan Jun Nie, Wei Chen

**Affiliations:** ^1^School of Life Science, Shanxi Normal University, Taiyuan, China; ^2^Xinjiang Institute of Plant Protection, Ürümqi, China; ^3^College of Agricultural Economics and Management, Shanxi Agricultural University, Taiyuan, China

**Keywords:** PGPF, *A. niger*, plant hormone signaling pathway, plant growth, tomato

## Abstract

**Introduction:**

Plant growth-promoting fungi (PGPF) play a fundamental role in plant development, such as nutrient acquisition and root growth. However, the growth promotion mechanisms regulated by *Aspergillus niger* are poorly characterized.

**Methods:**

We examined the growth-promoting effects of Aspergillus niger 129B on tomato plant root, stem, and leaf development through a combination of phenotype analyses and plant biomass measurement. Subsequently, molecular and genetic experiments were conducted to reveal the mechanism promoting root, stem, and leaf development.

**Results:**

It demonstrated that 129B significantly promoted the growth of tomato plants. Plant transcriptome and metabolome analysis revealed that this effect was associated with the plant hormone signaling pathway, particularly the expression of *SAUR32* and *PP2C72* genes. In addition, 129B could promote the development of root, stem, and leaf tissues by downregulating the expression of *SAUR32* and *PP2C72* genes. Importantly, we found that the promotion of tissue development may be attributed to the interaction between SAUR32 and PP2C72; the expression of SAUR32 proteins, which act as inhibitors of PP2C72 phosphatases, triggered root H^+^ efflux.

**Discussion:**

Our findings concluded that 129B-induced plant promotion is dependent on the interaction between SAUR32 and PP2C72, providing novel insights into beneficial plant–microbiome interactions.

## Graphical abstract

Role of *Aspergillus niger* in improving the tomato growth and inducing the SUAR32 and PP2C72 interaction.
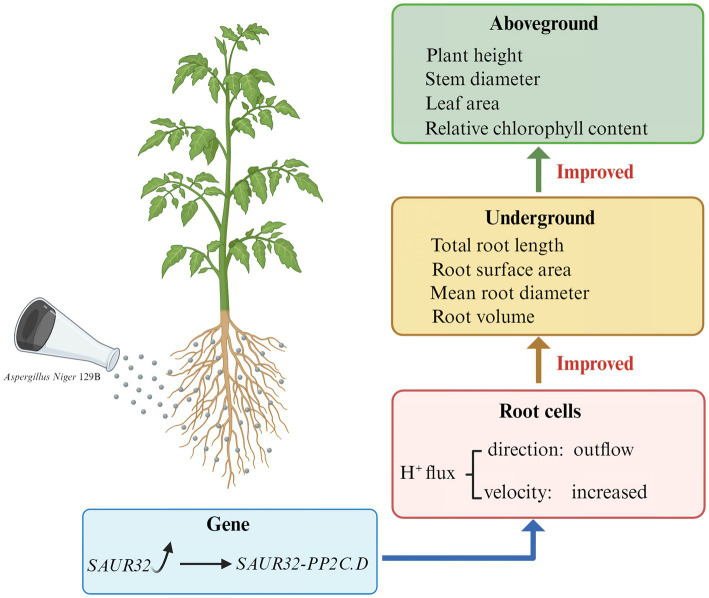

## Introduction

Microbes play a pivotal role in promoting plant growth and enhancing plant tolerance to abiotic and biotic stress. The consequences of these beneficial interactions include the emergence of specific plant-associated phenotypes (Oyserman et al., 2018), such as increased plant biomass, reduced pathogen growth, and enhanced plant growth and development (Batista et al., 2018). Microbiomes adhering to the surface or internal tissues of plant roots are selectively shaped by various plant-derived primary and secondary metabolites (Sasse et al., 2018; Canarini et al., 2019). Microbiomes reciprocate by sustaining plant growth and generating metabolites that regulate processes such as nutrient uptake and root elongation (Tracanna et al., 2021). Developing a comprehensive understanding of the genetic architecture of microbes that influence plant growth and how these interactions lead to specific plant-associated phenotypes is a pivotal focal point in current plant microbiology research (Gouda et al., 2018; Han et al., 2024). The promise is that plants can rely on specific members of the microbiome to improve growth and increase yields (Oyserman et al., 2021).

The plant growth-promoting microorganisms (PGPMs) are considered important biofertilizer resources that have profound impacts on ensuring crop growth, enhancing quality and quantity, improving soil properties, and controlling fungal pathogenesis, as well as preventing biotic stresses (Fu et al., 2024; Soumare et al., 2021). PGPMs can directly enhance plant nutrition acquisition from the soil and decompose organic matter through processes such as phosphate solubilization and nitrogen fixation (Alori et al., 2017). Additionally, PGPMs can mediate plant growth through producing exogenous phytohormones, such as auxin, abscisic acid (ABA), gibberellins, and ethylene (ET) (Ali and Khan, 2021). Some PGPMs can indirectly enhance plant growth by mitigating the deleterious effects of pathogens through systemic resistance induction, the production of repressive substances, and the improvement of plant nutrition (Gu et al., 2020). PGPMs commonly have more than one beneficial influence on plants through interacting with plant roots (Bruto et al., 2014). The growth stimulation potential of some economically available PGPMs has been studied through field experiments, including *Trichoderma harzianum*, *Aspergillus niger*, *Bacillus*, and *Pseudomonas*. However, the selection of new PGPMs with unique beneficial characteristics remains urgently needed. Recent studies have demonstrated that the genus Var*iovorax* can completely promote root growth caused by community-level microbes (Finkel et al., 2020); the genus *Massilia* can interact with soybean cultivars to promote seed oil accumulation (Han et al., 2024); and the species *Enterobacter* sp. SA187 can interact with *Arabidopsis thaliana* to cope with drought resilience and temperature stress (Andres et al., 2021); the genus *Streptomyces* can produce secondary metabolites to alleviate drought and salinity stress (Yang et al., 2023); and nd the species *Streptomyces* sp. NEAU6 can improve root growth and coleoptile growth in rice (Liu et al., 2022). The species *Trichoderma guizhouense* NJAU4742 produces the secondary metabolite anthranilic acid (2-AA), which promotes root development (Fu et al., 2025). Hence, the screening of new PGPMs and explanation of the molecular mechanisms of growth promotion have been proposed.

The root, stem, and leaf architecture is fundamental to improving plant growth and increasing grain yield. Specifically, changes in these architectures include plant height, stem diameter, overall root growth, root surface area, mean root diameter, root volume, and relative chlorophyll content (Mohamed et al., 2022). The root system directly interacts with microorganisms, which are essential for increasing the root surface area to continually absorb nutrients from the surrounding environment (Fu et al., 2025). The root formation process is controlled by cell wall adaptation that integrates auxin responses, root growth, and chemical signals. Plant root development depends on auxin-regulated genes in the auxin signaling pathway. SMALL AUXIN UP RNAs (SAURs) are primary auxin-responsive genes involved in the auxin signaling pathway to enhance plant root development and nutrient acquisition (Truskina et al., 2021). Previous research has confirmed that SAURs regulate plant developmental and physiological processes based on the acid growth theory. Expression of SAUR19, SAUR41, and SAUR63 genes is involved in regulating hypocotyl elongation and leaf size in *Arabidopsis*; the *SAUR39* gene governs the root meristem as a positive regulator in rice (He et al., 2021). Additionally, auxin signaling inhibits ATPase dephosphorylation through the SAUR-PP2C.D pathway, thereby regulating plant growth and development. The SAUR19 in *Arabidopsis* can inhibit PP2C.D phosphatases to activate the plasma membrane (PM)-localized P-type H^+^-ATPase (Spartz et al., 2014). Certain PGPMs have been shown to produce active substances that affect the auxin signaling pathway, thereby stimulating plant growth and development. For example, some bacterial species have been shown to activate auxin signaling within plants, which subsequently induces auxin-regulated gene expression, promoting plant growth (Ortiz-Castro et al., 2011). *Bacillus* spp. interacted with *Arabidopsis* and was able to influence auxin signaling to induce root development (Li et al., 2021). Additionally, the PGPMs of *Trichoderma* regulate *Arabidopsis thaliana* root morphogenesis via the auxin signaling pathway (Chen et al., 2024). These signaling pathways mediate plant growth and root development by secreting active substances. However, whether PGPMs can induce effects on the auxin signaling pathway in plants that regulate plant-associated phenotypes is unclear.

The genus *Aspergillus* is a filamentous ascomycete fungus widely used in industrial production and biotechnology research (Xin et al., 2023). It has also been identified as a plant growth-promoting fungus that effectively enhances crop quality and yield without causing environmental pollution (Mohamed et al., 2022). Some *Aspergillus* strains isolated from soil have been suggested to possess potential for interactions between *Aspergillus* and plants. The use of *Aspergillus* is a common strategy employed by researchers to enhance plant growth. Previous research has proved that some *Aspergillus* exert plant growth-promoting effects correlated with increases in tissue growth and plant biomass, for example, secreting secondary metabolites to promote plant growth and stimulate substances responsible for pathogen suppression (El-Maraghy et al., 2020); stimulating ion transport to increase vegetative growth, producing amino cephalosporanic acid acylase enzymes, and inducing plant immunity (Kriaa et al., 2015; Ismail et al., 2019); controlling fungal phytopathogens (Kour et al., 2019); and stimulating plant growth by facilitating nutrient acquisition and producing phytohormones, such as the small auxin-up RNA (SAUR). However, the plant growth promotion mechanism regulated by *Aspergillus* remains unclear. In this study, we investigated the impact of *Aspergillus niger* 129B on the growth and development of tomato plants. Tomatoes (*Solanum lycopersicum*) have been widely recognized as one of the most consumed vegetables in the global food industry. Ensuring a substantial tomato crop yield is crucial for meeting the growing global population’s need for nutritious food. Studying a tomato growth-promoting fungus probably meets the growing demand for food resources (Tayeb et al., 2024). In this study, we demonstrated that *Aspergillus niger* 129B induced phenotypic and biomass variability in tomato plants. This mechanism involved the expression of SAUR32 proteins, which act as inhibitors of PP2C72 phosphatases, thereby triggering root H^+^ efflux.

## Materials and methods

### Plant material

The experimental material consisted of tomato plants (*Solanum lycopersicum* L., cv. Tianfei 9) grown in a climate-controlled growth room at 25 ± 1°C, 65 ± 5% relative humidity, and a 16:8 h light:dark photoperiod. Tomato samples from 15-day-old seedlings of uniform growth and development were collected for conducting *Aspergillus niger* infection.

### Fungi material

The evolving lineages of *A. niger* in the experimental evolution assay were derived from the Shanxi Provincial Engineering Research Center for Microbial Application Technology, Shanxi, China, which we refer to here as the experimental strain *A. niger* 129B. The strain was cultivated on potato dextrose agar (PDA) at 28°C. *Escherichia coli* DH5α, grown in LB medium at 37°C, was used for plasmid propagation, while *Agrobacterium tumefaciens* AGL1, maintained on LB medium at 28°C, was used for plasmid transformation. Reagents for plasmid construction and real-time qPCR were obtained from Takara (China) to identify the experimental strain *A. niger* 129B. Spores of *A. niger* 129B were collected from PDA cultures incubated at 28°C for 7 days and subsequently cultured in corn steep liquor at a concentration of 1 × 10^−6^ CFU/mL. This spore suspension was used for *A. niger* infection of tomato roots.

### Fungi treatment

For the tomato root experiments, 15-day-old tomato seedlings (one seedling per pot) were transferred to containers containing natural soil. Immediately after transplantation, the base of the tomato root was infected with 25 mL of fungal suspension or with 25 mL of aseptic water as a control. Subsequently, the fungi suspension-treated seedlings were continuously irrigated with 25 mL of the solution every other day until the 45th day, while the control seedlings, which did not receive *A. niger* 129B treatment, were irrigated with 25 mL of water until the 45th day. The tomato plants were cultivated in a growth chamber with a controlled temperature (26 ± 1°C) and a photoperiod of 16 h of light and 8 h of darkness. For each treatment, three independent experiments were carried out. Samples from the fungal treatment were used for morphological traits and molecular analyses.

### Measurement of plant height, stem thickness, and root growth

The aboveground growth condition of the tomato between the control and *A. niger* 129B was photographed on days 0, 7, 14, 21, 28, and 35, respectively. The plants with uniform growth were selected from each treatment on the 35th day of *A. niger* incubation to measure their height and stem thickness using a ruler.

We measured root morphological traits after carefully washing the roots with DI water and removing any adhering moisture. The determination of root architecture parameters followed the methodology of Gao et al. (2023). Tomato seedling roots with uniform growth were scanned using an Epson Perfection V850 Pro scanner (Epson, Tokyo, Japan). The resulting scanned images were analyzed using specialized root analysis software (WinRHIZO 2016, Regent Instruments Corporation, Shanghai, China). Root growth-related parameters, including total root length, total root surface area, average root diameter, and root volume, were determined for each treatment on the 15th, 30th, and 45th days of *A. niger* incubation. Three biological replicates were measured for each treatment.

### Measurement of chlorophyll content

The tomato leaf growth conditions between the control and *A. niger* 129B were photographed on days 0, 7, 14, 21, 28, and 35, respectively. Chlorophylls of the 35th day post-treatment (35 dpt) leaf of tomato were extracted with 80% acetone. Chlorophyll content in leaves was determined at 663 nm via a spectrophotometer (Evolution 300, Thermo Fisher Scientific, United States) using the BioTek Synergy H4 Hybrid Multi-Mode Microplate Reader. Each treatment had three biological replications.

### Measurement of H^+^ flux using NMT

Tomato plants at the fourth leaf stage, 14 dpt with *A. niger* 129B, were washed with deionized (DI) water, and root tissues were fully immersed in experimental solution (0.1 mM CaCl2, 0.3 mM 2-(N-morpholino) ethanesulfonic acid, pH = 6) for 30 min of equilibration. Then, H^+^ fluxes at the tomato root apex were recorded using the non-invasive microtest technique (NMT, 100S-SIM-XY, Xuyue Sci & Tech Co., Ltd., Beijing, China) according to the previously reported method (Wang et al., 2022). Continuous transient H^+^ efflux was monitored at the meristematic region (500 μm from the root tip) and recorded for 5 min. Each measurement was conducted using five biological replicates. Data analysis was conducted using imFluxes V2.2 software.

### Transcriptome analysis

Fungal infection samples and control samples treated with aseptic water at 30 dpt were stored at −20°C until further transcriptome analysis. Transcript assay total RNA extraction using the TransZol Up Plant RNA Kit (Beijing Transgenbiotech Co., Ltd., Beijing, China) for Illumina^®^ was performed according to previous research (Wu et al., 2023). The mRNA product was purified from 3 μg of RNA using poly-T-oligo-attached magnetic beads. Subsequently, the purified mRNA fragments were fragmented into small pieces using a proprietary fragmentation buffer at elevated temperatures. First-strand cDNA was synthesized through reverse transcription using random oligonucleotide primers SuperScript II, followed by second-strand cDNA synthesis using DNA Polymerase I and RNase H. TailingMix and RNA Index Adapters added for terminal repair incubation were selectively enriched using Illumina PCR Primer Cocktail in a 15-cycle PCR reaction. PCR amplification products of cDNA fragments were purified using the AMPure XP system and dissolved in EB solution. The sequencing library generated from the PCR product was denatured by heating. Finally, looping through the splint oligo sequence facilitated the formation of single-stranded circular DNA (ssCir DNA), completing the final library. The transcriptomic analysis of gene functional annotations was performed as described previously (Wu et al., 2023), including Gene Ontology (GO), eggNOG, Kyoto Encyclopedia of Genes and Genomes (KEGG) databases, GO enrichment analysis, KEGG pathway enrichment analysis of differentially expressed genes, and KEGG Orthology (KO) analysis of unigenes. All the above measurements were performed with five replications.

### Metabolite data analysis

Tomato seedlings, 30 days post-transplantation (dpt), were used for the metabolomics assay. The experimental group was infected with 25 mL of fungal suspension, while the control seedlings, which did not receive *A. niger* 129B treatment, were irrigated with 25 mL of aseptic water. All samples were stored at −80°C until further metabolomics analysis. Eighty milligrams of the thawed samples were obtained to add a pre-cooled methanol/acetonitrile/aqueous solution (2:2:1, v/v), and then quickly frozen with liquid nitrogen to grind into a fine powder. Subsequently, the powder was added to 1,000 μL of a methanol/acetonitrile/H2O mixture (2:2:1, v/v/v), and the mixture was centrifuged to collect the supernatant for metabolomics analysis. The significance of differences in metabolomes was analyzed using the partial least squares discriminant analysis (PLS-DA) methodology and a t-test, as well as fold change (FC) analysis (Wu et al., 2024). Differentially expressed metabolites were determined using principal component analysis (PCA). Differentially expressed metabolites (DEMs) were determined with the parameters of variable importance of projection (VIP) in the PLS-DA model ≥1 and fold-change ≥1.5 or ≤0.67. The Kyoto Encyclopedia of Genes and Genomes (KEGG) databases were used to annotate the differential metabolites and identify associated metabolic pathways. Five biological replications were measured for each treatment.

### qRT-PCR validation of RNA-seq data

The expression levels of eight differentially expressed genes were selected to validate the reliability of the RNA-seq data using quantitative real-time PCR (qRT-PCR) technology (CFX96 Real-Time System, Bio-Rad, California, United States). The reaction system of the Real-Time PCR System, using SYBR Green as the fluorescent dye, was employed according to the manufacturer’s protocol. The PCR amplification system for each sample had a total volume of 25 μL, consisting of 1 μL of cDNA, 1 μL of gene-specific primers, 10.25 μL of RNase-free water, 0.25 μL of Rox Dye (50×), and 12.5 μL of 2 × PerfectStart Green qPCR SuperMix (TransGen Biotech, China). The PCR reaction system comprised an initial denaturation step at 95°C for 5 min, followed by 40 cycles of 10 s at 95°C, 15 s at 55°C, and 20 s at 72°C. The validation of RNA-seq data consisted of three biological replicates with three technical replicates. *Solanum lycopersicum*. SL3.0.51.genomme.fa (S1) was the internal control gene.

### RNA extraction and qRT-PCR analysis

The tomato plant at 45 dpt with *A. niger* 129B was available for analysis of gene expression in root, stem, and leaf. Total RNA extraction from each sample of tomato root, stem, and leaf utilizing the TransZol Up Plant RNA Kit (Beijing Transgenbiotech Co., Ltd., Beijing, China) was performed according to the manufacturer’s instructions. Quantitative reverse transcription PCR (qRT-PCR) was performed using the PerfectStart Uni RT & qPCR Kit (Beijing Transgenbiotech Co., Ltd., Beijing, China) on a Stratagene Mx3000P Real-Time PCR system (Stratagene, CA, United States) in accordance with the manufacturer’s protocols. The primers are as follows: PP2C72-F: TAAGGAACTGTGATGGTAGAG, PP2C72-R: GCAAGAACAAGCAGATGAC; SAUR32-F: GCATCCCAAAGGGGTGTCTT, SAUR32-R: GGTTATGATGTCCGGTGGCA; S1Actin-F: GGAATGGGACAGAAGGAT, S1Actin-R: CAGTCAGGAGAACAGGGT. The PCR amplification system for each sample had a total volume of 20 μL, consisting of 1 μL of cDNA, 0.8 μL of gene-specific primers, 7.8 μL of RNase-free water, 0.4 μL Universal Passive Reference Dye (50×), and 10 μL of 2 × PerfectStart Green qPCR SuperMix (TransGen Biotech, China). The PCR reaction protocol included an initial denaturation step at 95°C for 5 min, followed by 40 cycles of 10 s at 95°C, 15 s at 55°C, and 20 s at 72°C. S1 was utilized as the internal reference control. The relative expression levels of genes SAUR32 and PP2C72 were calculated using the 2^−ΔΔCT^ method. Each sample was represented by three biological replicates with three technical replicates.

### Subcellular localization

The experimental tobacco (*Nicotiana benthamiana*) used in this research was cultivated in a greenhouse at 24°C and 37% relative humidity, with a 16-h light/8-h dark photoperiod. Moreover, 15-day-old tomato seedlings were used to obtain the cDNA sequences of the genes SUAR32 and PP2C72 for vector construction. The coding regions of SAUR32 and PP2C72 were inserted into the NcoI/SpeI sites of pCAMBIA1302. The subcellular localization of SUAR32 and PP2C72 fused to the GFP fluorescent reporter was performed in *N. benthamiana* leaf protoplasts in accordance with the protocol described previously (Wang et al., 2011). Briefly, protoplast cells from the third and fourth leaves, counting from the top of each tobacco plant, were isolated and transformed via the PEG-mediated protoplast transfection method (Wang et al., 2011). Full-length cDNA was fused with GFP in PGBKT7-SAUR32 and PGADT7-PP2C72, which were individually transiently transformed into the protoplasts of *N. benthamiana* leaves. After incubation for two nights, GFP fluorescence images were captured using a fluorescence confocal microscope (Dragonfly CR-DFLY-505; Suzhou Anqin Air Technology Corporation, Suzhou, China).

### Yeast two-hybrid assay

Yeast two-hybrid (Y2H) assays were performed in accordance with the manufacturer’s protocol. The complete coding sequences of the *SAUR32* and *PP2C72* genes were amplified from the Tianfei 9 variety using 15-day-old tomato seedlings and primers as described above. The coding regions of SAUR32 and PP2C72 were inserted between the NdeI/BamHI sites of pGADT7 and between the NdeI/BamHI sites of pGBKT7. The resulting PCR fragments were sequenced to validate their reliability and then integrated into pGBKT7 or pGADT7 vectors using double-restriction enzyme digestion with NdeI/BamHI. The resulting recombinant bait and prey plasmids were co-transformed into the yeast Y2H strain and selected on SD-Leu-Trp media at 30°C for 3 days. Subsequently, a selective single colony was inoculated into liquid SD-Leu-Trp medium and incubated overnight at 30°C. Eventually, the overnight cultures were serially diluted into three concentrations and spotted on SD-Leu-Trp and SD-Leu-Trp-His media, followed by incubation at 30°C for 3 days.

### Bimolecular fluorescence complementation

The coding regions of SAUR32 and PP2C72 from 15-day-old tomato seedlings were used for the vector construct. Bimolecular fluorescence complementation analysis was conducted as previously described (Kudla and Bock, 2016). Briefly, the coding regions of SAUR32 and PP2C72 were inserted between the NdeI/BamHI sites of pGADT7 and pGBKT7, respectively. pSPYCE(M)-SAUR32, pSPYNE173-PP2C72, pSPYCE(M), and pSPYNE173 were introduced into *Agrobacterium tumefaciens* strain GV3101 and co-transformed into approximately 5-week-old *N. benthamiana* leaves via agroinfiltration. GFP fluorescence signals were detected and imaged 3 days post-infiltration using a fluorescence confocal microscope (Dragonfly CR-DFLY-505; Suzhou Anqin Air Technology Corporation, Suzhou, China).

### Statistical analysis

All experimental data were analyzed using a minimum of three independent biological replicates with SPSS (IBM SPSS Statistics 25). A one-way ANOVA with Tukey’s *post hoc* test was applied to compare the means between the controls and treatments, with a *p*-value of < 0.05.

## Results

### Plant growth-promoting effect of *Aspergillus niger* 129B

**Figure 1:**
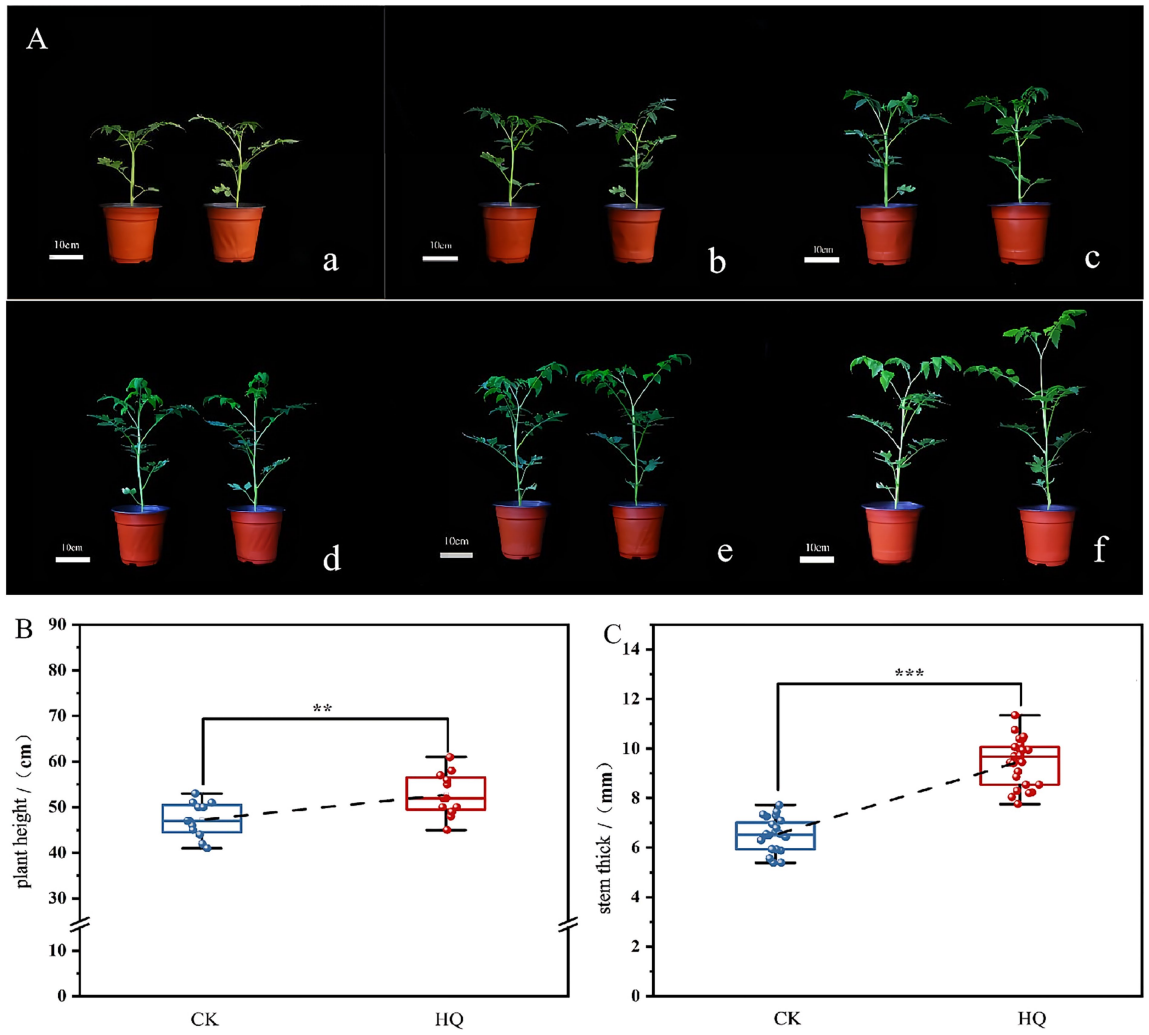
Effects of fungi treatment on stem growth. *A. niger* 129B treatment was conducted by irrigating the tomato plant with 25 mL of the solution once every other day. Controls included tomato plants without *A. niger* 129B treatment instead of irrigating 25 mL of aseptic water once a day. **(A)** Morphology (scale = 10 cm) of tomato after *A. niger* 129B treatment under different day treatments. **(a–f)** Represent the comparison of tomato growth between the control and *A. niger* 129B on day 0, day 7, day 14, day 21, day 28, and day 35, respectively (left: control group, right: *A. niger* 129B treatment group). **(B)** Effects of *A. niger* 129B treatment on plant height after 35 dpt. **(C)** Effects of *A. niger* 129B treatment on stem thickness after 35 dpt. Values are represented as means ± SD of three replicates. Significant differences were based on one-way ANOVA with Tukey’s *post hoc* test **(B,C)** (*p* < 0.05).

**Figure 2:**
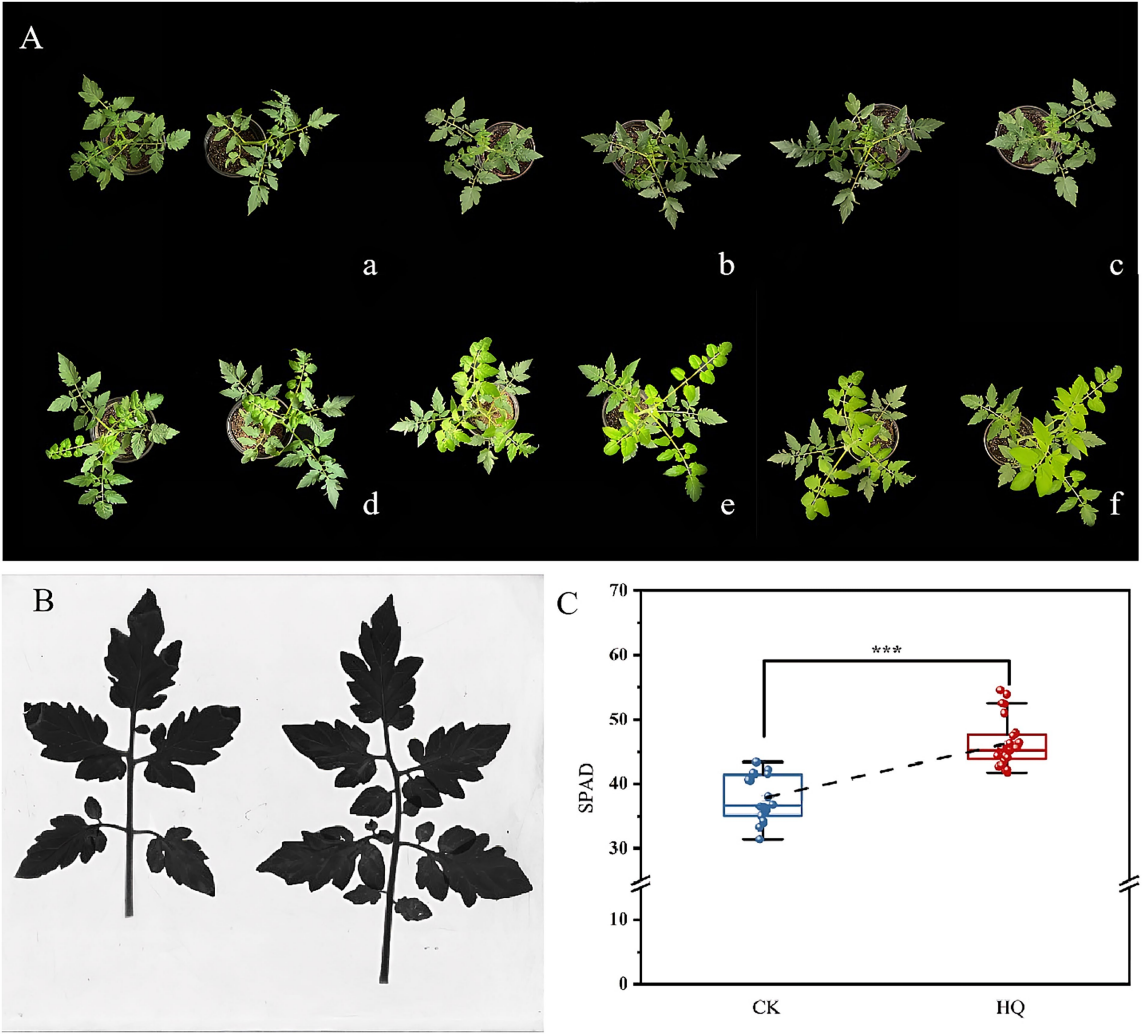
Effects of fungi treatment on leaf growth. The control and treatment were the same as above. **(A)** Effects of *A. niger* 129B on the morphology (scale = 10 cm) of tomato leaf growth under different day treatments. **(a–f)** Represent the comparison of tomato leaf growth between the control and *A. niger* 129B on day 0, day 7, day 14, day 21, day 28, and day 35, respectively (left: control group, right: *A. niger* 129B treatment group). **(B)** Leaf scan image of *A. Niger* 129B on tomato leaves after 35 dpt (left: control group, right: *A. niger* 129B treatment group). **(C)** Effects of *A. Niger* 129B on tomato relative chlorophyll content after 35 dpt. Data are expressed as the mean ± SD of three replicates. Significant difference by one-way ANOVA with Tukey’s *post hoc* test (*p* < 0.05).

**Figure 3:**
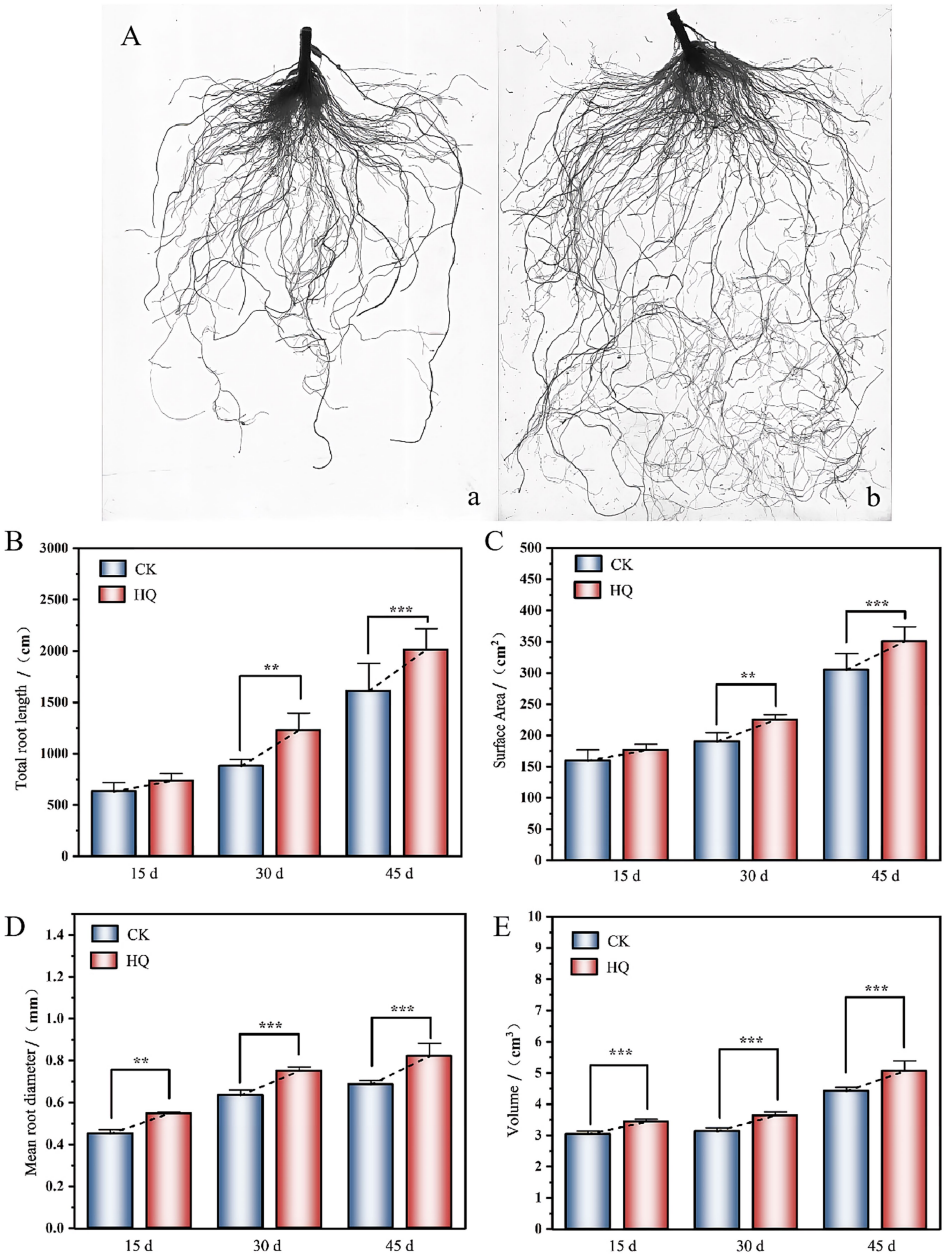
Effects of fungi treatment on root growth. **(A)** Visual display of tomato roots under *A. niger* 129B treatment at 45 dpt (**a**: control group, **b**: *A. niger* 129B treatment group). **(B–E)** Root growth treated with *A. niger* 129B after 15 days, 30 days, and 45 days treatment (**B**: total root length; **C**: surface area; **D**: mean root diameter; and **E**: root volume). Values are represented as means ± SD of three replicates. Significant differences based on one-way ANOVA with Tukey’s *post hoc* test (*p* < 0.01).

To determine the influence of *A. niger* 129B infection on tomato plant growth across different day treatments, we measured morphological characteristics related to plant growth, including plant height, stem diameter, total root length, root surface area, mean root diameter, root volume, relative chlorophyll content, and both aboveground and underground growth conditions. *A. niger* 129B infection significantly promoted tomato plant growth by increasing plant height, stem diameter and aboveground growth (Figure 1A). These effects were statistically significant at 35th day post-treatment (35 dpt) compared to the water-treated control. Specifically, stimulation with *A. niger* 129B resulted in an 11.64% increase in plant height (52.75 cm) and a 45.40% increase in stem diameter (9.48 mm) at 35 dpt compared to control values of 47.25 cm and 6.52 mm, respectively (Figures 1B,C). Irrigation with *A. niger* 129B also led to notable aboveground leaf growth and increased relative chlorophyll content. At 35 dpt, *A. niger* 129B treatment significantly improved tomato leaf parameters, showing a 25.47% increase in relative chlorophyll content (46.3) compared to the control (36.9) (Figure 2). In terms of underground growth, *A. niger* 129B inoculation enhanced root tissue growth compared to control plants at 15th, 30th, and 45th days post-treatment (dpt). At 45th dpt, total root length increased by 16.38, 39.26, and 24.83%; root surface area by 9.61, 15.45, and 13.00%, respectively, mean root diameter by 21.42, 18.40, and 19.66%; and root volume by 11.43, 13.74, and 12.60%, respectively, compared to the control treatment. However, at 15th dpt, *A. niger* 129B did not show statistically significant differences compared to the control (Figure 3).

### H^+^ fluxes measurement analysis

**Figure 4:**
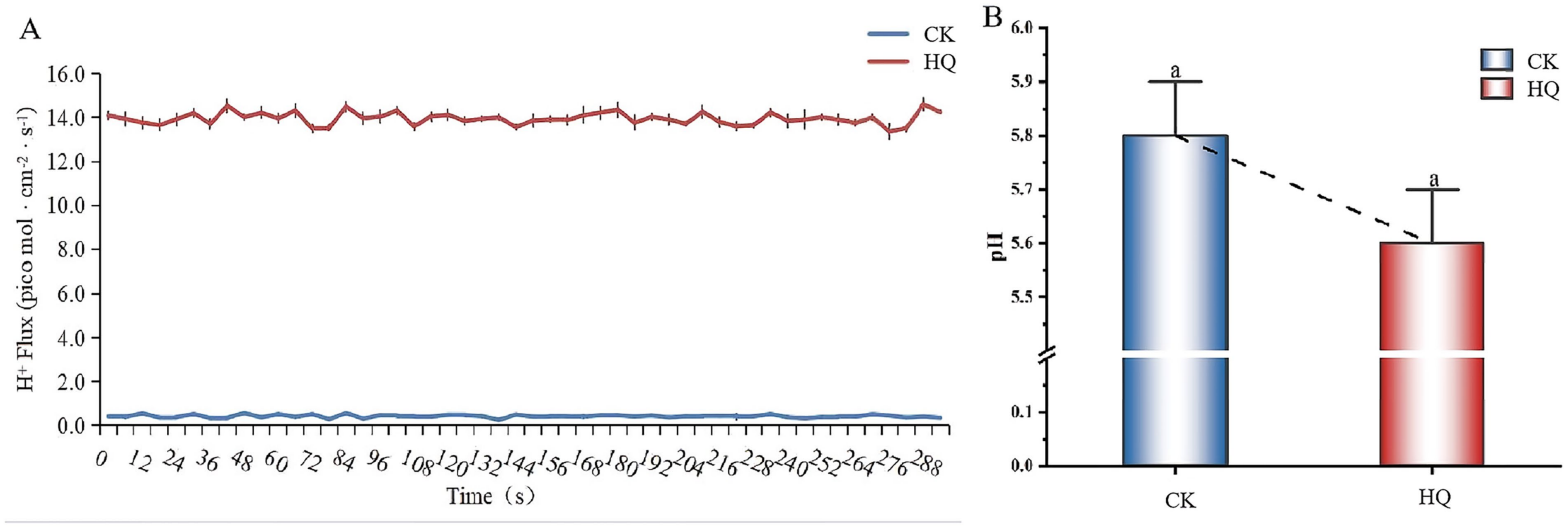
H^+^ fluxes in tomato root tips and pH value in tomato root surface of *A. niger* 129B infection at 14 dpt. **(A)** The H^+^ flux in tomato roots. **(B)** The pH value in the tomato root surface region.

The H^+^ flux recordings showed a highly significant efflux after *A. niger* 129B infection at 14 dpt (Figure 4). The H^+^ efflux reached 14 pmol·cm^−2^ s^−1^ in tomato roots treated with *A. niger* 129B, while control roots showed minimal H^+^ efflux at 0.4 pmol·cm^−2^ s^−1^ (Figure 4A). The pH value at the root surface of *A. niger* 129B-infected plants decreased by 0.2 compared to the control (Figure 4B). This increased H^+^ efflux was driven by H^+^-ATPases in the plasma membrane, which contributed to enhanced tomato growth after *A. niger* 129B infection.

### Combined transcriptomic and metabolomic analysis of tomato plants

**Figure 5:**
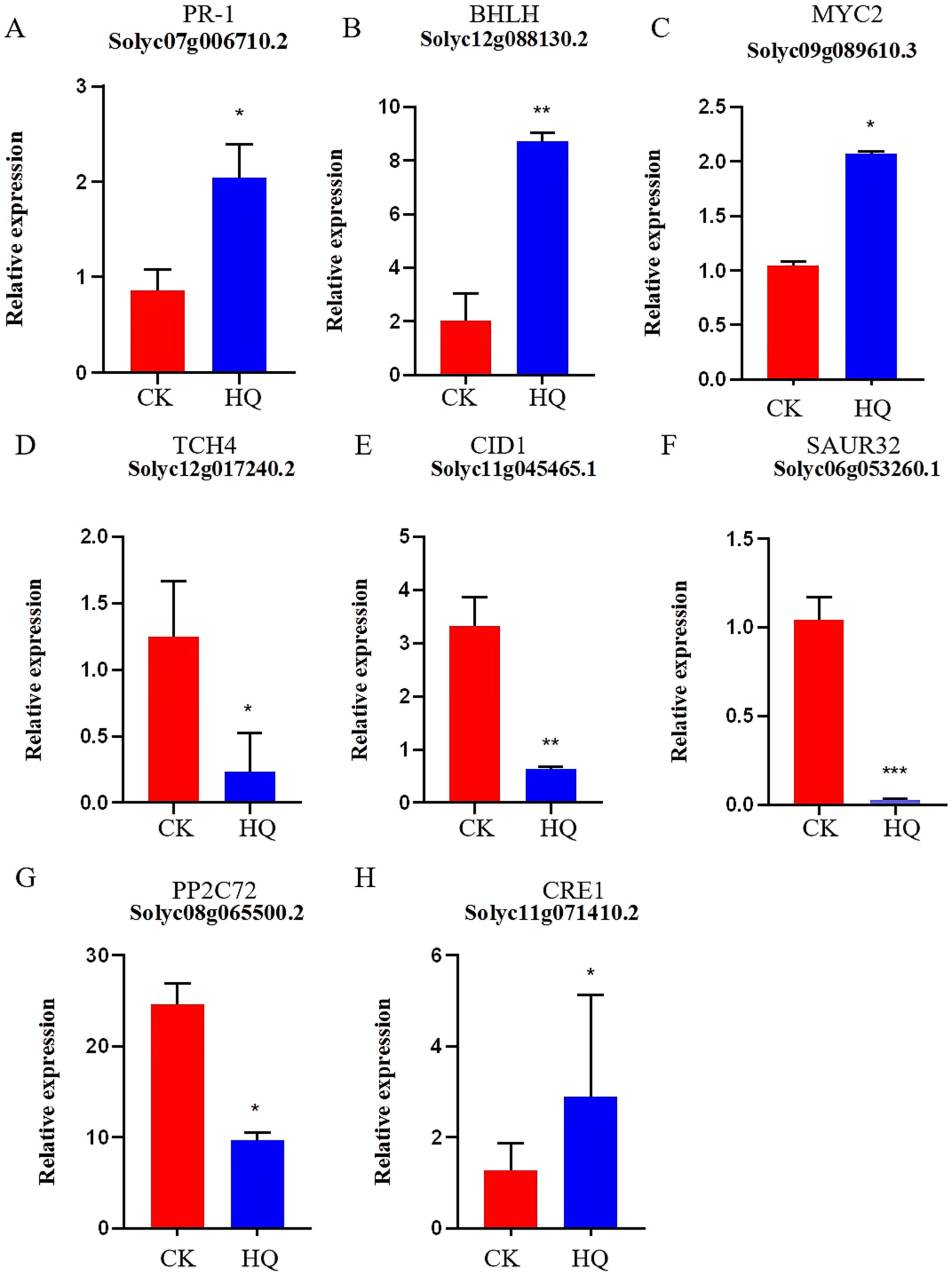
Histogram of gene expression. HQ means tomato treated with *A. niger* 129B after 14 dpt; CK represents tomato treated with aseptic water after 14 dpt. **(A–H)** Represent PR-1 (Solyc07g006710.2), *BHLH* (Solyc12g088130.2), MYC2 (gene_Solyc09g089610.3), TCH4 (gene_Solyc12g017240.2), GID1 (gene_Solyc11g045465.1), SAUR32 (Solyc06g053260.1), PP2C72 (gene_Solyc08g065500.2) and CRE1 (Solyc11g071410.2), respectively.

**Figure 6:**
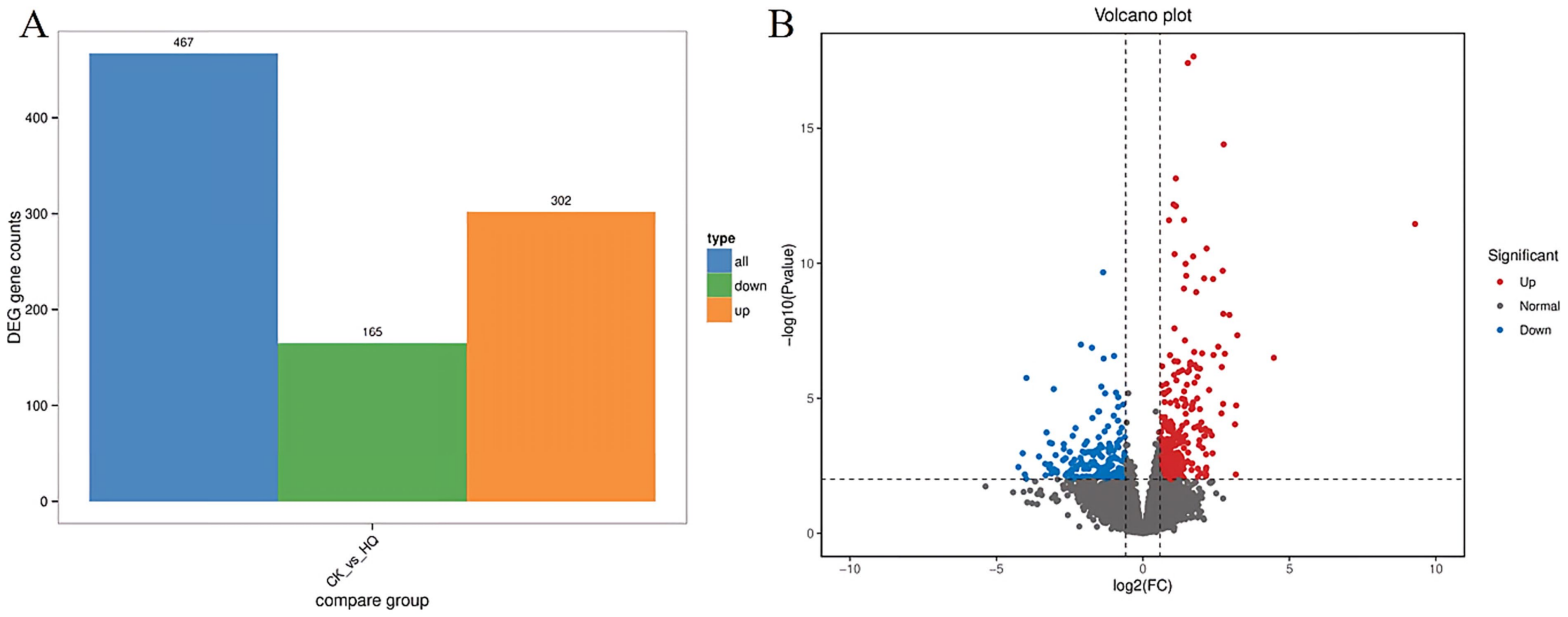
Differentially expressed genes (DEGs) between the *Aspergillus niger* 129B and control samples. CK was tomato treated with aseptic water after 14 dpt, and HQ means tomato treated with *Aspergillus niger* 129B after 14 dpt. DEGs are determined based on |log2(Fold Change)| ≥1.5 and *p* < 0.05. **(A)** Statistical histogram of differential genes under different treatments **(B)** Volcano map of different.

**Figure 7:**
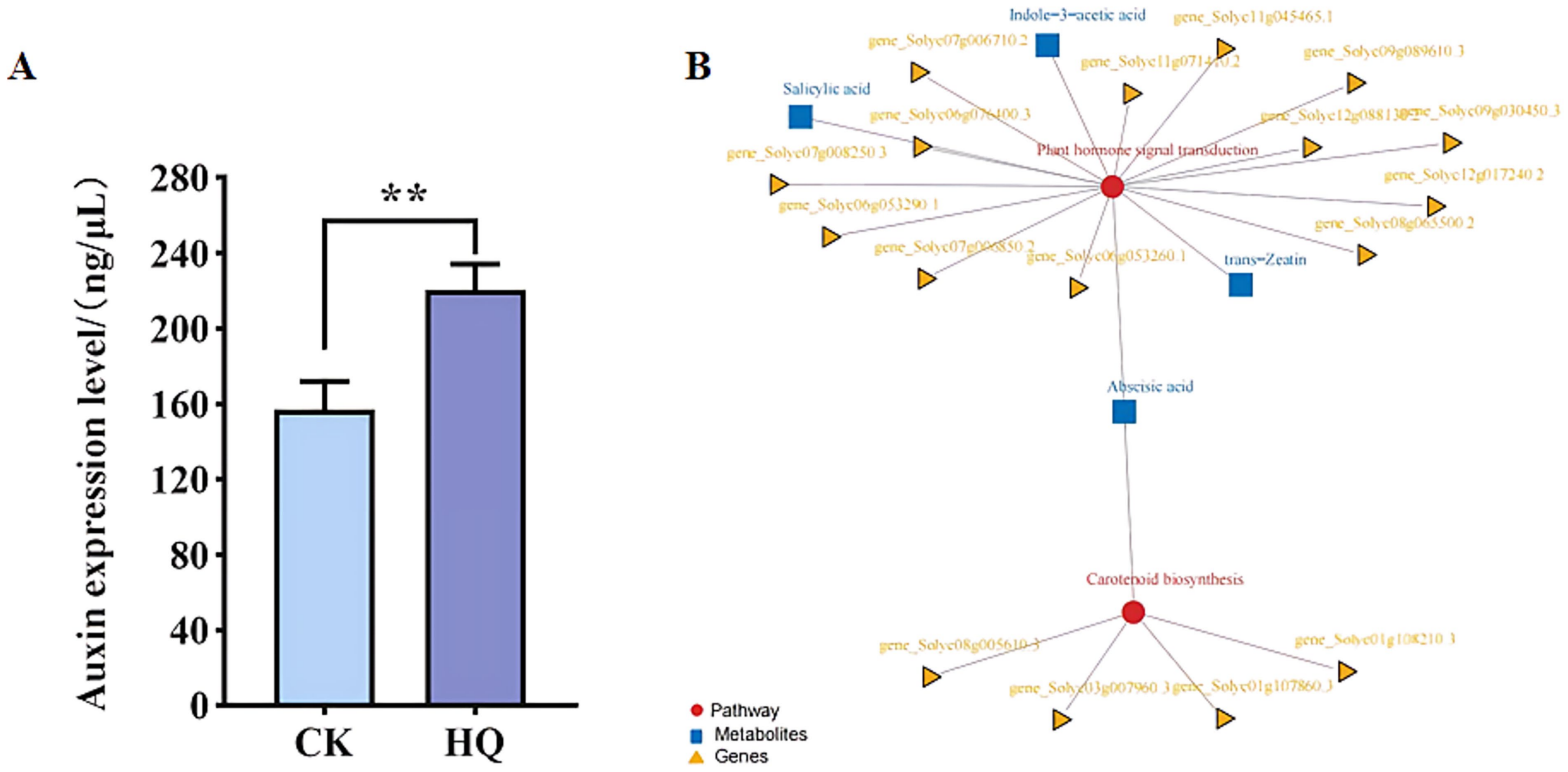
Correlation analysis of transcriptomic and metabolomic data of *Aspergillus niger* 129B. **(A)** Pathway and differential gene/metabolite network of tomato treated with *Aspergillus niger* 129B and aseptic water. Circles represent pathways; triangles represent genes; boxes represent metabolites. **(B)** The concentrations of IAA induced by *Aspergillus niger* 129B. CK means tomato treated with aseptic water after 14 dpt, and HQ means tomato treated with *Aspergillus niger* 129B after 14 dpt. **(A)** pSPYCE(M)-SAUR32 and pSPYNE173-PP2C72 constructs. **(B)** pSPYCE(M)-SAUR32 + pSPYCE (M) as control. **(C)** pSPYNE173-PP2C72 + pSPYNE173 as control. Fluorescence, Bright field and Merge indicate images of fluorescence, bright field and merge, respectively.

Transcriptome analysis techniques have been developed in the tomato plant in response to *A. niger* 129B infection in our research. The expression levels of 13 differentially expressed genes (DEGs) were randomLy validated to assess the reliability of the real-time qPCR experiment, and 8 of these genes passed the differential abundance criteria in both RNA-seq and qPCR analysis (Table 1 and Figure 5). The expression of tomato genes identified in control groups and *A. niger* treatment was used to identify the DEGs based on |log2(Fold Change) | ≥1.5. A total of 467 significant DEGs were detected in transcriptomic analysis. Three hundred and two DEGs of them showed different degrees of upregulation, while 165 of them performed downregulation in the *A. niger* 129B treatment compared with the control (Figure 6). Small auxin up RNA (SAUR)-mediated cell elongation and protein phosphatase class 2C (PP2C), involved in regulating plant growth, showed significant downregulation (Figure 5). The combined transcriptomic and metabolomic analysis indicated that SAUR32 interacts with the PP2C72, which regulates the plant signal transduction under *A. niger* 129B infection (Figure 7A). Moreover, *A. niger* 129B induced the plant to produce IAA, with a maximum yield of 203.64 ng/μL after 14 days of incubation, compared to the control, which yielded 157.05 ng/μL (Figure 7B).

**Table 1:** Statistics of transcriptome sequencing of tomato plant under *A. niger* 129B infection and control conditions after 14 dpt.

Sample	ReadSum	BaseSum	Total reads	Mapped reads	Uniq mapped reads	Multiple map reads
CK1	42,152,756	12,625,695,524	84,305,512	75,471,106	73,729,955	1,741,15
CK2	39,770,037	11,912,842,078	79,540,074	76,066,670	74,102,196	1,964,47
CK3	36,945,206	11,067,007,242	73,890,412	66,226,917	64,725,484	1,501,43
HQ1	37,838,133	11,334,992,074	75,676,266	70,548,738	68,653,347	1,895,39
HQ2	38,200,980	11,443,686,916	76,401,960	73,741,555	72,058,759	1,682,79
HQ3	41,497,689	12,430,442,764	82,995,378	78,874,103	77,125,684	1,748,41

CK means control, CK1, CK2, and CK3 represent three technical replication. HQ means *A. niger* 129B infection, HQ1, HQ2, and HQ3 represent three technical replications.

### Expression analysis of *SAUR32* and *PP2C72* from tomato plants

**Figure 8:**
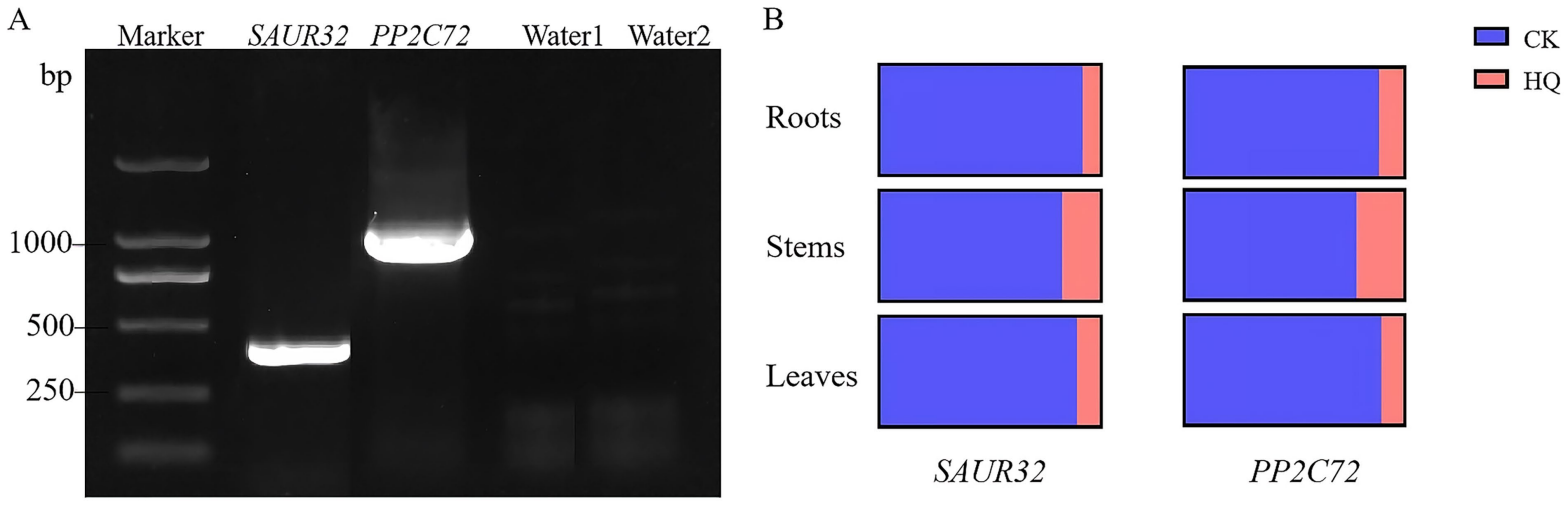
The expression patterns of *SAUR32* and *PP2C72* genes from a tomato plant with the *A. niger* 129B infection at 14 dpt. **(A)** The amplification of genes *SAUR32* and *PP2C72*. **(B)** qRT-PCR of gene *SAUR32* and *PP2C72* expression in tomato root, stem, and leaves. The region of the image means the control with aseptic water, and the pink region of the image represents the *Aspergillus niger* 129B infection.

The *A. niger* 129B treatment for tissue expression analysis of *SAUR32* and *PP2C72* revealed its presence at 15th dpt. The *SAUR32* and *PP2C72* gene transcript levels were identified utilizing a qRT-PCR experiment (Figure 8A). The results showed that the expression of the *SAUR32* and *PP2C72 genes* in all test tissues affected by *A. niger* 129B infection was affected. When subjected to *A. niger* 129B treatment, the transcript levels in tomato roots, stems, and leaves exhibited downregulation, with the lowest value observed after 14 days of *A. niger* 129B infection (Figure 8B).

### Subcellular localization of SAUR32 and PP2C72

**Figure 9:**
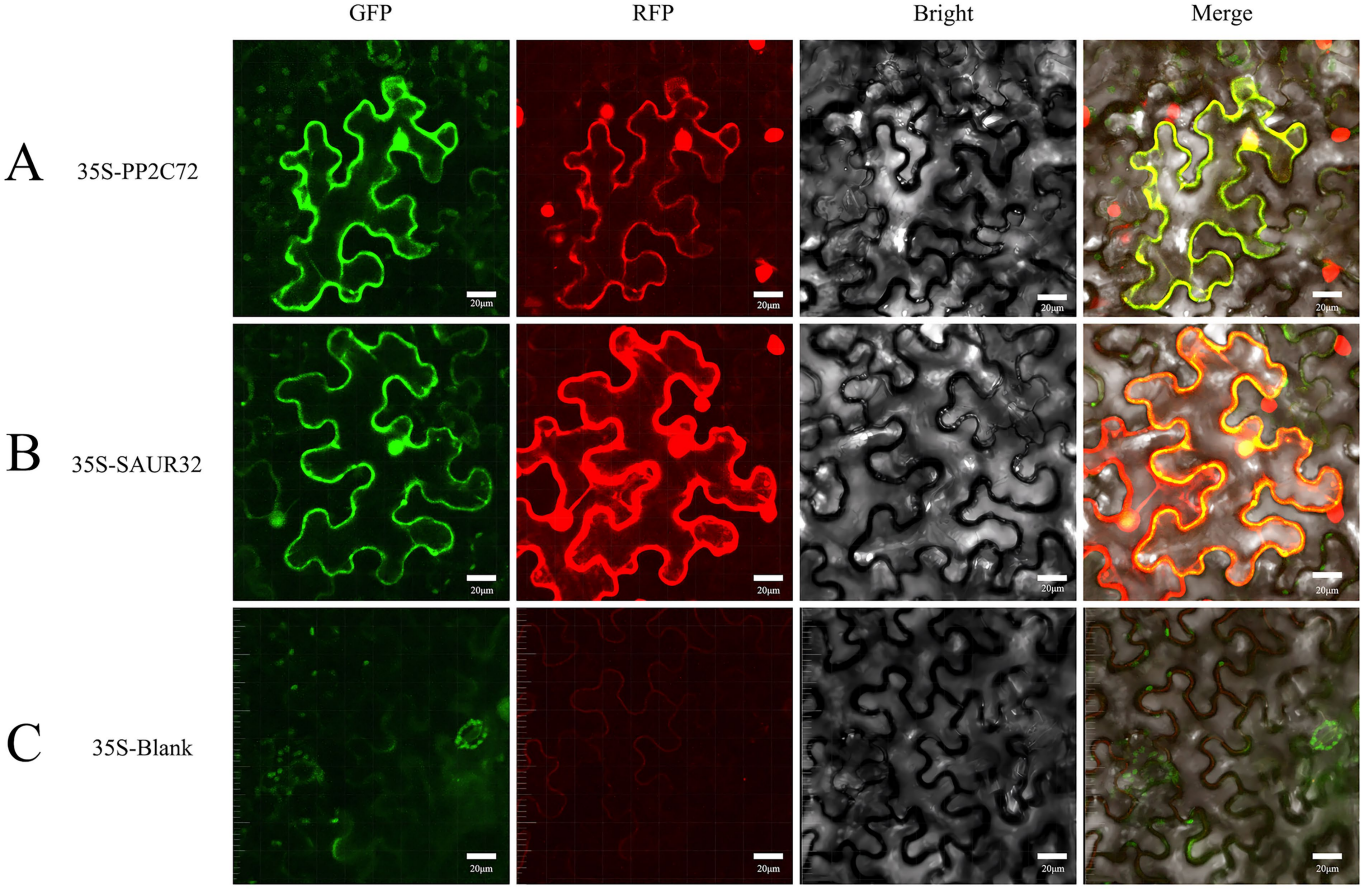
Subcellular localization of PP2C72 and SAUR32 in *Nicotiana benthamiana* leaf epidermis cells. After incubation for 2 days, the fluorescent signals in the tobacco leaf epidermis cells expressing were recorded using confocal microscopy. The panels in this image have 20 μm scale bars. **(A)** pCAMBIA1302-PP2C72 constructs. **(B)** pCAMBIA1302-SAUR32 constructs. **(C)** The empty vector pCAMBIA1302.

To investigate the subcellular localization of SAUR32 and PP2C72, transient expression of SAUR32-GFP, PP2C72-GFP, SAUR32-GRFP, and PP2C72-RFP fusion proteins was performed by injecting the constructs into *N. benthamiana* leaves for 2 days. The construct containing the 35S promoter was used as a blank control. Fluorescent signals from the SAUR32-GFP, PP2C72-GFP, SAUR32-RFP, and PP2C72-RFP fusion proteins were observed in the transformed protoplasts and were detected in both the nucleus and cell membrane (Figure 9). These findings strongly suggest that SAUR32 and PP2C72 were localized in the nucleus and cell membrane.

### SAUR32 interacts with PP2C72 under yeast two-hybrid analyses

**Figure 10:**
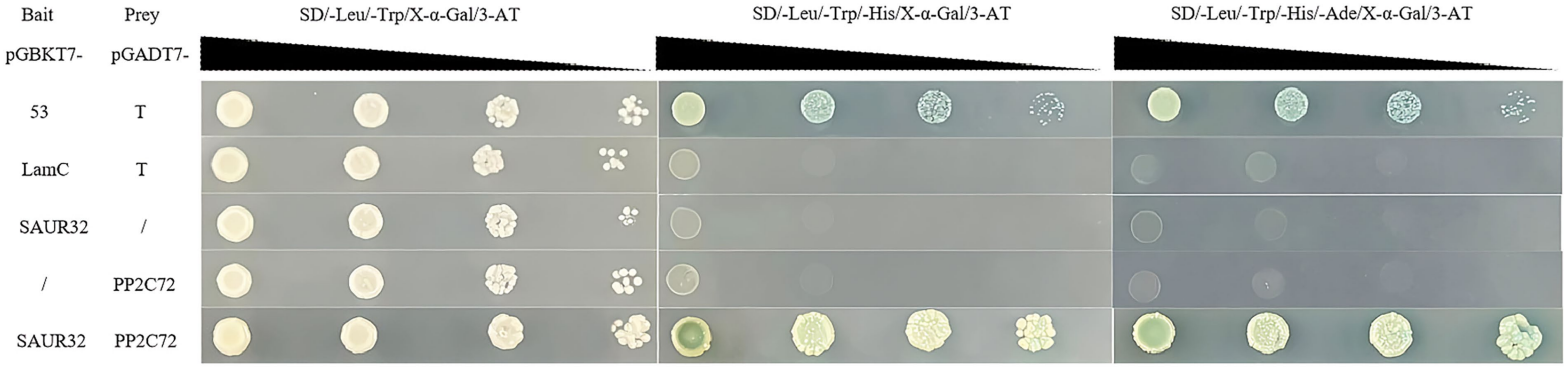
Tomato SAUR32 interacts with PP2C72 under yeast two-hybrid (Y2H) analyses. Positive (+) and negative (−) controls are pGADT7-53 + pGBKT7-T and pGADT7-Lam + pGBKT7-T, representing known interacting and non-interacting.

Here, we verified the interaction between SAUR32 and PP2C72 using yeast two-hybrid (Y2H) analysis. In our research, we observed that PP2C72-pGADT7 and pGBKT7-SAUR32 cotransformed yeast AH109 were grown in medium at 30°Cfor 3 days. Research findings indicate that the cotransformed positive yeast turned blue on the selective medium (SD-Trp-Leu-His-Ade) containing x-α-gal. The PP2C72-pGADT7 fusion with pGBKT7-SAUR32 in AH109 exhibited growth under specific conditions (Figure 10). These results indicated that SAUR32 interacted with PP2C72.

### SAUR32 interacts with PP2C72 with BiFC assays

**Figure 11:**
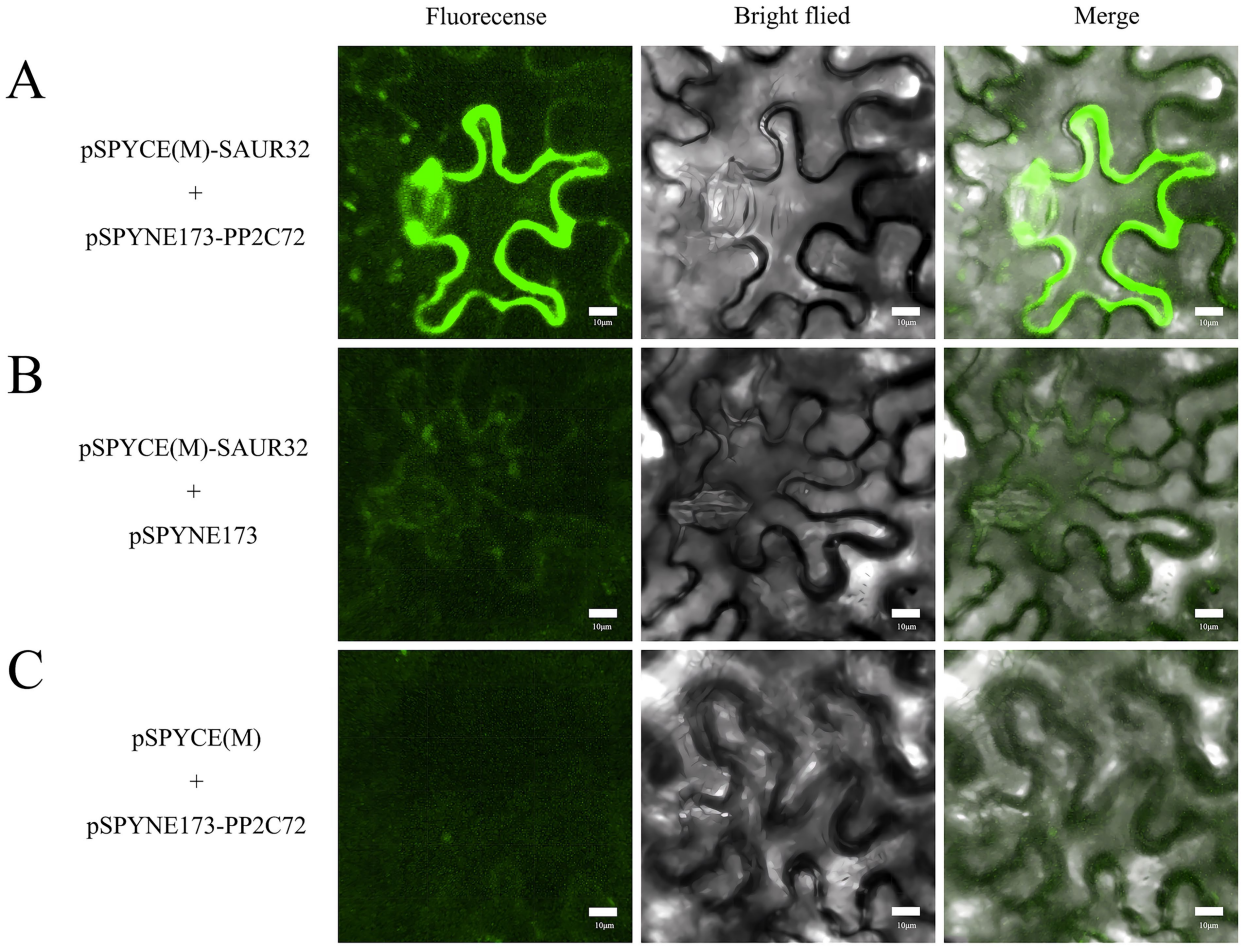
Bimolecular fluorescence complementation in *Nicotiana benthamiana*. Green fluorescence with a full length means the interaction between SAUR32 and PP2C72. Bar 10 μm. **(A)** pSPYCE(M)-SAUR32 and pSPYNE173-PP2C72 constructs. **(B)** pSPYCE(M)-SAUR32 + pSPYCE(M) as control. **(C)** pSPYNE173-PP2C72 + pSPYNE173 as control. Fluorescence, Bright field and Merge indicate images of fluorescence, bright field and merge, respectively.

To elucidate the SAUR32 and PP2C72 interaction *in vivo*, the study was performed using bimolecular fluorescence complementation (BiFC) assays by infiltration of pSPYCE(M)-SAUR32, pSPYNE173-PP2C72, or pSPYCE(M), pSPYNE173 into *N. benthamiana* leaf samples. Nuclear green fluorescence detected signals from *N. benthamiana* leaves that were, respectively, introduced with pSPYCE(M)-SAUR32 and pSPYNE173-PP2C72 constructs via the *Agrobacterium* (GV3101) infiltration method, while no fluorescence signal was present in live plants injected with pSPYCE(M) and pSPYNE173 (Figure 11). The BiFC assays confirmed that SAUR32 interacts with PP2C72.

## Discussion

The filamentous ascomycete fungus *A. niger* has been reported to promote plant development and root growth, acting as a plant growth-promoting fungus (Mohamed et al., 2022); however, its growth-promoting mechanisms have not been fully characterized. In this research, we found that *A. niger* 129B significantly improved the above-ground and underground growth conditions (Figures 1–3). Notably, infection with *A. niger* 129B promoted the tissue development of the root, stem, and leaf in tomato, including plant height, stem diameter, total root length, root surface area, mean root diameter, root volume, and relative chlorophyll content, which are important contributors in plant regulation of various processes. The above morphological characteristics in tomato plants increased at the 35th day post-treatment compared with the sterile water treatment. The identified *A. niger* strain of PGPF was evaluated for its biocontrol potential against tomato *Fusarium* wilt; however, it is present in limited amounts, promoting plant growth (Anwer and Khan, 2013; Galeano et al., 2021). This research confirmed that *A. niger* 129B significantly improved the plant growth of tomato plants, compensating for the lack of plant growth-promoting fungal mechanisms through *A. niger* stimulation.

Several beneficial functions of plant growth-promoting fungi have been reported to mediate various aspects of plant growth. The previous studies showed that *A. niger* linked to plant development and root elongation utilizing as a plant growth-promoting fungus, for example, enhanced the growth of healthy and infected tomato plants (Anwer and Khan, 2013); changed the phosphate solubilization and maize growth (Yin et al., 2015); promoted the growth of native forage grass (Galeano et al., 2021); improved the photosynthetic pigment in tomato plants (Dufossé, 2006); increased the contents of carotenoids, chlorophyll b, total soluble proteins carbohydrates, total phenols and free proline, the POD and PPO activity in tomato plant (Mohamed et al., 2022). In our study, transcriptomics and metabolomics analysis revealed that the plant IAA content exhibited relatively high percentages compared with the control (Figure 6A). The plant hormone IAA has been reported to improve plant growth in agriculture (Cao et al., 2019; Fu et al., 2025).

There are several aspects by which PGPMs can improve plant growth, including nitrogen metabolism, phosphate solubilization, and IAA production of auxin (Bergelson et al., 2021). The SAURs in auxin are well-known plant growth-related auxin-responsive genes that regulate plant tissue development (Zhou et al., 2025). The expression of SAUR family members in different plants plays vital roles in growth-promoting microbiomes, which improve plant growth through root architecture, hypocotyl elongation, the formation of apical hooks, and leaf growth (Anastasios et al., 2022; Jia et al., 2024). Therefore, SAUR32 may contribute to the growth-promoting effect of *A. niger* 129B in tomato plants. These results are in agreement with previous research. For example, *Beauveria bassiana* colonization on tomato plants increased the expression level of the drought tolerance-related gene *SAUR32* (Mohamed et al., 2022); *arbuscular mycorrhizal* fungus inoculation observed the DEGs related to auxin-responsive protein SAUR32 in the root system of *Camellia sinensis* L. (Chen et al., 2021); and inoculation with beneficial *Suillus luteus* upregulated auxin-responsive protein SAUR32 expression in *Scots* pine plants (Wen et al., 2025).

SAUR32 contributed to the beneficial traits of PGPMs in promoting plant growth, and this growth mechanism is likely associated with the acid growth theory (Lin et al., 2021). Auxin promotes apoplastic acidification in hypocotyls, with growth regulation mediated by TIR1/AFB-Aux/IAA nuclear auxin signaling. Auxin-triggered, TIR1/AFB-dependent expression of SAUR proteins inhibits PP2C.D phosphatases. This inhibition can activate H^+^-ATPase activity and regulate pavement cell morphogenesis, thereby enhancing root thermomorphogenesis (Lin et al., 2021; Chen et al., 2024). In our study, the stimulation of plant growth-promoting effects by strain *A. niger* 129B involves the SAUR32 proteins acting as inhibitors of PP2C72, which implicates the activation of the H^+^ pump. This activation accelerates the efflux of H^+^, resulting in a decrease in pH. Previous studies have demonstrated that SAUR19 and SAUR36 family proteins, as positive effectors, interact with PP2C.D phosphatases to modulate PM H^+^-ATPase activity, thereby promoting hypocotyl cell expansion and regulating hypocotyl elongation in *Arabidopsis* (He et al., 2021). Although colonization by *A. niger* 129B led to the downregulation of *SAUR32* gene expression in tomato plants, these findings suggest that SAUR32 negatively regulates cell expansion and that its suppression promotes root elongation under *A. niger* 129B inoculation. This result was in agreement with previous findings, where *SAUR32* overexpression hindered apical hook development and led to shortened hypocotyls in *Arabidopsis* plants (Park et al., 2007).

Plants have been proposed to exhibit root development, allowing them to cope with environmental variations. In this regard, PGPMs are considered essential for influencing root growth and development by regulating cell expansion and differentiation. In *Arabidopsis*, *Pseudomonas* spp. can stimulate root primordium formation and enhance its numbers of early-stage (Ortiz-Castro et al., 2011); members of the genus *Trichoderma* remarkably promoted root primordium formation and its number of late-stage (Chen et al., 2024; Fu et al., 2024); and *Bacillus amyloliquefaciens* improved the periodic root development and increased its numbers of early-stage (Li et al., 2021). Additionally, *Sinomonas gamaensis* NEAU-HV1 significantly promotes the root growth of various plants (Fu et al., 2024). Previously, *A. niger* has been confirmed to improve plant development by acting as a plant growth-promoting fungus. However, the impact of *A. niger* on root development is poorly characterized in previous studies. Here, we verified that the root development parameters, including total root length, root surface area, mean root diameter, and root volume, were significantly increased under stimulation with *A. niger* 129B (Figure 3). The reason for the root elongation treated with *A. niger* 129B may contribute to the downregulation of the *SAUR32* gene in the auxin response (Figure 7). Moreover, the downregulation of the SAUR32 gene in the root system may explain how SAUR32 proteins act as inhibitors of PP2C72, thereby contributing to the root growth of the tomato plant induced by *A. niger* 129B inoculation.

To the best of our knowledge, the canonical TIR1/AFB-SAUR-PP2C.D auxin signaling pathway is crucial for root development (Ortiz-Castro et al., 2011). The root formation strongly suggested that these two families of proteins act antagonistically to control cell expansion. TIR1/AFB-dependent expression of SAUR proteins leads to the repression of PP2C.D phosphatase activity. This inhibition prevents plasma membrane H^+^-ATPase activation, regulating apoplastic pH changes to govern root cell expansion (Lin et al., 2021; Chen et al., 2024; Fu et al., 2024). *Pseudomonas aeruginosa* enhanced the degradation of Aux/IAA protein degradation and requires the auxin signaling cascade IAA-ARF; the compound BiAux accumulation in roots can bind to a specific TIR1 site to increase coreceptor formation between IAA and TIR1/AFB for root growth (Ortiz-Castro et al., 2011); *Sinomonas gamaensis* NEAU-HV1 could improve root development independently of the auxin receptor TIR1/AFB2 (Fu et al., 2024). Here, we identified that *A. niger* 129B stimulation can promote the interaction between SAUR32 and PP2C72. The regulatory effect of *A. niger* on SAUR32 expression may induce *PP2C72* downregulation, thereby promoting root elongation. This novel regulatory mechanism may be attributed to the SAUR32 genes, which acquire cell-type-specific functions by engaging the PP2C72-H^+^-ATPase cellular machinery under *A. niger* 129B infection. The *A. niger* is functionally activated in the canonical TIR1/AFB-SAUR-PP2C.D auxin signaling pathway (Lin et al., 2021). Therefore, we hypothesize that *A. niger* probably migrates through the cell membrane and enters the nucleus to regulate the plant growth process. A similar growth hypothesis has been proposed for *Sinomonas gamaensis* NEAU-HV1, which mediates the IAA-ARF interaction to improve plant growth (Fu et al., 2024). Additionally, the regulatory modules of SAUR32-PP2C72 antagonism accelerate the efflux of H*^+^*, resulting in a decrease in the pH of acidification (Figure 11). These findings suggest that the *A. niger*-mediated noncanonical auxin signal systems, which regulate cell expansion and root growth, provide a comprehensive explanation for how auxin controls growth and developmental processes through intracellular auxin-signaling pathways. Therefore, the present findings suggest that the intracellular auxin-signaling pathways sustain PM H^+^-ATPase activation in cells where auxin-mediated cell expansion of the tomato root occurs, collectively explaining the acid growth theory of root growth induced by *A. niger*.

## Conclusion

Our study clearly demonstrated that *Aspergillus niger* 129B can improve plant development and promote root elongation. The 129B-induced growth promotion is dependent on the interaction between SAUR32 and PP2C72; the expression of SAUR32 proteins, which act as inhibitors of PP2C72 phosphatases, triggers root H^+^ efflux, stimulating plant growth. To the best of our knowledge, the research is the first to explain how *A. niger* can specifically promote plant growth through the SAUR32-PP2C72 module.

## Data Availability

The original contributions presented in the study are included in the article/supplementary material. Further inquiries can be directed to the corresponding authors.
